# Pharmacodynamics of Linezolid Plus Fosfomycin Against Vancomycin–Resistant *Enterococcus faecium* in a Hollow Fiber Infection Model

**DOI:** 10.3389/fmicb.2021.779885

**Published:** 2021-12-14

**Authors:** Shuaishuai Wang, Huiping Liu, Jun Mao, Yu Peng, Yisong Yan, Yaowen Li, Na Zhang, Lifang Jiang, Yanyan Liu, Jiabin Li, Xiaohui Huang

**Affiliations:** ^1^Department of Basic and Clinical Pharmacology, School of Pharmacy, Anhui Medical University, Hefei, China; ^2^Anhui Province Key Laboratory of Major Autoimmune Diseases, Anhui Institute of Innovative Drugs, School of Pharmacy, Anhui Medical University, Hefei, China; ^3^Department of Infectious Diseases, The First Affiliated Hospital of Anhui Medical University, Hefei, China

**Keywords:** hollow-fiber infection model, linezolid, fosfomycin, vancomycin-resistant *Enterococcus faecium*, antibiotics resistance, virulence

## Abstract

The optimal therapy for severe infections caused by vancomycin-resistant *Enterococcus faecium* (VREfm) remains unclear, but the combination of linezolid and fosfomycin may be a good choice. The 24-h static-concentration time-kill study (SCTK) was used to preliminarily explore the pharmacodynamics of linezolid combined with fosfomycin against three clinical isolates. Subsequently, a hollow-fibre infection model (HFIM) was used for the first time to further investigate the pharmacodynamic activity of the co-administration regimen against selected isolates over 72 h. To further quantify the relationship between fosfomycin resistance and bacterial virulence in VREfm, the *Galleria mellonella* infection model and virulence genes expression experiments were also performed. The results of SCTK showed that the combination of linezolid and fosfomycin had additive effect on all strains. In the HFIM, the dosage regimen of linezolid (12 mg/L, steady-state concentration) combined with fosfomycin (8 g administered intravenously every 8 h as a 1 h infusion) not only produced a sustained bactericidal effect of 3∼4 log_10_ CFU/mL over 72 h, but also completely eradicated the resistant subpopulations. The expression of virulence genes was down-regulated to at least 0.222-fold in fosfomycin-resistant strains compared with baseline isolate, while survival rates of *G. mellonella* was increased (*G. mellonella* survival ≥45% at 72 h). For severe infections caused by VREfm, neither linezolid nor fosfomycin monotherapy regimens inhibited amplification of the resistant subpopulations, and the development of fosfomycin resistance was at the expense of the virulence of VREfm. The combination of linezolid with fosfomycin produced a sustained bactericidal effect and completely eradicated the resistant subpopulations. Linezolid plus Fosfomycin is a promising combination for therapy of severe infections caused by VREfm.

## Introduction

Enterococcus is an important conditionally pathogenic gram-positive bacteria and is a major cause of hospital-acquired infections, which can cause urinary tract infections, soft tissue infections, and also serious infections such as bacteremia (Tan et al., 2020). What’s worse, although vancomycin is the “first-line treatment” for severe enterococcal infections, its inappropriate use has led to the development of vancomycin-resistant enterococci (VRE) infections (Kutkowska et al., 2019). In the face of VRE infection (especially *Enterococcus faecium*), clinical monotherapy is often ineffective and even associated with high mortality and clinical failure rates (Snyder et al., 2016; Hemapanpairoa et al., 2019; Goić-Barišić et al., 2020). Thus, it is urgent to find antibiotic combinations to treat vancomycin-resistant *Enterococcus faecium* (VREfm) infections.

Linezolid has entered clinical practice as one of the most dependable agents for the treatment of VREfm infections (Kalfopoulou and Huebner, 2020; Ramos et al., 2020). However, studies have shown that the standard dosing of 600 mg linezolid intravenously twice a day for severe infections caused by VREfm could predispose cases of clinical treatment failure (Hemapanpairoa et al., 2019). Even, some studies indicated that increasing the dose and treatment time of linezolid may lead to greater hematologic toxicity and the development of resistance during linezolid therapy (Ye et al., 2018; Jiang et al., 2021). Considering the limitations of linezolid monotherapy, a combination of drugs may be a good approach. Fosfomycin, an old antibiotic, which has a unique mechanism of bactericidal activity by inhibiting an early stage of bacterial cell wall biosynthesi (Dijkmans et al., 2017). Given fosfomycin’s unique mechanism of action, the safety and no cross-resistance with other agents, it may provide a useful option for the treatment of patients with multidrug-resistant gram-positive bacterial infections, including VREfm (Zhanel et al., 2018; Petrosillo et al., 2019; Abbott et al., 2020). The previous studies of our group confirmed that linezolid combined with fosfomycin could effectively inhibit vancomycin-resistant and -sensitive enterococcus and prevent enterococcus resistance (Qi et al., 2019; Jiang et al., 2021). However, previous studies were limited by constant antibiotic concentration over 24 h, making it difficult to guide rational clinical administration, much less explain the development of resistance from a quantitative perspective.

More recently, the hollow-fiber infection model (HFIM), which has been widely used to explore how to rationally use antibiotics and reduce bacterial resistance, can provide valuable information about the interactions between antibiotics, bacteria, and host (Nielsen and Friberg, 2013; Burgos and Rodvold, 2019; Zhao et al., 2020; Agyeman et al., 2021). In the HFIM, Tsuji et al. (2012) found that the front-loaded dose of linezolid against VREfm could provided the maximal bacterial reductions of about 3 log_10_ CFU/ml, but the front-loaded regimens did not inhibit the expansion of resistant subpopulations against VREfm with resistant alleles. However, there has been no report on the pharmacodynamic activity of linezolid combined with fosfomycin against VREfm in the HFIM. With the development of drug resistance, the clinical efficacy of combination drugs depends not only on the killing of total bacteria, but also on the suppression of resistant subpopulations. Therefore, we need to explore the potential efficacy of the combination regimen against VREfm in the HFIM in terms of both total bacterial load and resistant subpopulations.

As resistance develops, uncovering the indirect effects of antibiotic resistance on virulence may be particularly important in high bacterial load infections (Bulman et al., 2015). Studies have shown that antimicrobial resistance may not only incur fitness costs, but also lead to changes in bacterial virulence *in vivo* (Scortti et al., 2018). In some cases, the mechanisms of fosfomycin resistance reduced the virulence of the bacteria that present fosfomycin resistance (Díez-Aguilar and Cantón, 2019). Moreover, publications have shown that the possible interaction between bacterial virulence and drug resistance in VREfm (Ghorbanzadeh et al., 2020). However, the specific relationship between fosfomycin resistance and bacterial virulence in VREfm has not been reported in the literature. These virulence factors, such as the enterococcal surface protein (*esp*) and adhesin of collagen from *Enterococcus faecium* (*acm*), enable enterococcus to successfully colonize the host and participate in biofilm formation (Strateva et al., 2016; Gao et al., 2018; Gaca and Lemos, 2019; M Campos et al., 2020). Therefore, these two virulence genes were used as breakthroughs to explore the relationship between fosfomycin resistance and bacterial virulence in VREfm from a quantitative perspective. Additionally, *Galleria mellonella* has been further used in bacterial virulence studies because it has an innate immune system similar to that of mammals (Tsai et al., 2016; Kavanagh and Sheehan, 2018; Meyer et al., 2019).

In this study, 24-h static-concentration time-kill (SCTK) study was used for preliminary investigation of the pharmacodynamic activity of linezolid in combination with fosfomycin against three VREfm strains. On this basis, the HFIM was used to further evaluate the efficacy of the combination regimen for bactericidal and resistance suppression against selected isolates over 72 h. In addition, the relationship between fosfomycin resistance and bacterial virulence in VREfm was further investigated by survival rate of *G. mellonella* and quantitative expression of virulence genes.

## Materials and Methods

### Bacterial Isolates

Three clinical isolates of VREfm (NO.1, NO.2, and NO.3) were isolated from the urine of different patients admitted to the First Affiliated Hospital of Anhui Medical University. All strains were identified by the automated VITEK-2 system (BioMerieux, Marcy l’Etoile, France). Vancomycin-resistant Enterococcus ATCC 51299 was used as the quality control strain. In addition, these strains were not specifically isolated for this research but were part of the routine hospital laboratory procedure. This study was approved by the First Affiliated Hospital of Anhui Medical University institutional review board.

### Antimicrobials and Media

Linezolid, Fosfomycin and Vancomycin were purchased from the National Institute for Food and Drug Control of China (Beijing, China). Mueller-Hinton broth supplemented with calcium and magnesium (CAMHB, Oxoid, United Kingdom; 25.0 mg/L Ca2+, 12.5 mg/L Mg2+) and Mueller-Hinton agar (MHA, Oxoid, United Kingdom) were used for all experiments and Brain Heart Infusion agar (BHIA, Oxoid, United Kingdom) were only used for the susceptibility testing of Vancomycin. In addition, all media to which fosfomycin was added also contained 25 mg/L glucose-6-phosphate (Sigma-Aldrich).

### *In vitro* Susceptibility Testing and Mutation Frequency

Minimum inhibitory concentrations (MICs) of all antibiotics were determined using the agar dilution method according to Clinical and Laboratory Standards Institute guidelines (CLSI, 2019). Briefly, Mueller-Hinton agar and Brain Heart Infusion agar plates containing a series of two-fold concentration increments of each agent were prepared. The agar plates containing fosfomycin needed to add glucose-6-phosphate and made the final concentration 25 mg/L. Then, ∼10^5^ colony forming units (CFU) of bacterial cells were inoculated with these plates and incubated at 37^°^C for 18–24 h. The MIC was defined as the lowest drug concentration in that inhibited the visible growth of colonies. Vancomycin-resistant Enterococcus ATCC 51299 was used as the quality control strain for these experiments. MIC determinations were performed in triplicate for each strain.

An overnight incubation in CAMHB of three isolates was subsequently serially diluted and plated on drug-free MHA plates to estimate the total bacterial burden and also on drug-containing (3 × baseline MIC) MHA plates to estimate the resistant subpopulation burden. The ratio of the resistant subpopulation burden to the total bacterial burden provided an estimate of the drug resistance frequency within the total population.

### *In vitro* Static-Concentration Time-Kill Study

*In vitro* activities of fosfomycin and linezolid alone and in combination were initially assessed using the SCTK. In short, overnight cultures of three VREfm isolates were adjusted in turbidity using supplemented CAMHB to achieve a ∼10^8^ CFU/mL starting inoculum, approximating a high-bacterial-burden infection. Fosfomycin was studied at 128, 256, and 512 mg/L (normal clinical serum range ∼150 to 500 mg/L; Zhao et al., 2017) and linezolid of 12 mg/L. These concentrations were studied in monotherapy and combination therapy against all three isolates. Samples were collected at 0, 2, 4, 6, 8, 12, and 24 h, serially diluted in 0.9% saline and then plated onto MHA plates for viable counting. All tests were performed in triplicate. Additivity and synergy were defined as 1–2 log_10_ CFU/mL and ≥2 log_10_ CFU/mL greater reductions with the combination compared to the most active single drug in the combination, respectively. Furthermore, bacteriostatic and bactericidal activities were defined as <3 log_10_ and ≥3 log_10_ reductions in CFU/mL at 24 h, respectively, relative to the starting inoculum.

### Hollow Fiber Infection Model

The HFIM, which was previously used and described in detail (VanScoy et al., 2014), was modified slightly in the present study to simulate the pharmacokinetic/pharmacodynamic of these antimicrobials. VREfm NO.2 was selected as a representative strain for the HFIM because it showed the strongest synergistic bactericidal effect in the SCTK. CAMHB was pumped from a central compartment through a hollow fiber cartridge (C2011, FiberCell Systems, Inc., Frederick, MD, United States) before being returned to the Central compartment. Drugs was administered into the central compartment by using a programmable peristaltic pump (Ismatec, Cole-Parmer, Inc., Barrington, IL, United States). Fresh media was continuously supplied and removed from the central compartment at an appropriate rate to simulate the average human half-lives of the antimicrobials. Linezolid in this study was a constant concentration of 12 mg/L, derived from the approximate median of the plasma trough concentration distribution of linezolid measured in 150 patients receiving 600 mg of linezolid every 12 h at the time of initial treatment monitoring assessment (Cattaneo et al., 2016). For the fosfomycin administration regimens, the elimination half-life (*t*_1/2_) of 4.8 h was simulated and a two-compartment pharmacokinetic model for fosfomycin *in vitro* concentration data was fitted using Phoenix WinNonlin software (Sauermann et al., 2005).

Prior to each experiment, bacterial from an overnight growth on CAMHB was suspended and added to each model to obtain a starting inoculum of ∼10^8^ CFU/ml and allowed resistant subpopulations to be present at baseline. The model apparatus was maintained at 37^°^C throughout the experiment. The following dosing regimens were evaluated in the HFIM: 1. Fosfomycin at 8 g every 8 h (q8h), (area under the free-drug concentration–time curve from time 0–8 h [fAUC_0–8_], 1,266.8 mg⋅h/L; maximum concentration of free-drug in serum [fCmax], 370.0 mg/L); 2. Fosfomycin at 12 g q8h (fAUC_0–8_, 1,901.0 mg⋅h/L; fCmax, 554.9 mg/L); 3. Fosfomycin at 8 g × 2 q8h on day 1 (fAUC_0–8_, 2,538.4 mg⋅h/L; fCmax, 744.0 mg/L) followed by 8 g q8h on days 2,3 (fAUC_0–8_, 1,266.8 mg⋅h/L; fCmax, 370.0 mg/L); 4. Linezolid of 12 mg/L (continuous infusion); and 5. Fosfomycin at 8 g q8h plus linezolid of 12 mg/L. In addition, each dose of fosfomycin was administered as a 1-h infusion. Control experiments with no drug treatment were run under the same conditions as used for the treatment arms, except for fewer sampling time points.

### Pharmacokinetic-Pharmacodynamic Analysis

In each experiment, serial bacterial samples (0.5 mL) were obtained from the extracapillary compartment at 0, 2, 4, 6, 8, 12, 24, 32, 40, 48, 56, 64, and 72 h and diluted in 0.9% saline. Serial dilutions were then plated on both drug-free and drug-containing (3 × baseline MIC) MHA plates to enumerate total and resistant subpopulation, respectively. The plates were incubated at 37^°^C for 24 h (total population) or 72 h (resistant subpopulation). The lower limit of detection was 100 CFU/ml for drug-free and 10 CFU/ml for drug-containing MHA plates. Bacterial isolates were recovered from fosfomycin-supplemented plates at the end of the experiments, and the fosfomycin MICs were reestimated to investigate whether the mutants had an elevated fosfomycin MIC.

Pharmacokinetic samples (0.5 mL) were collected at predetermined timepoints from the central reservoir of the HFIM. All samples were immediately stored at −80^°^C until analyzed using a validated bioassay method. Fosfomycin concentrations were determined after appropriate dilution using a previously described biological assay that utilized *Escherichia coli* ATCC 25922 as an indicator organism (VanScoy et al., 2016). The standard curve of fosfomycin showed a good linearity in the concentration range of 50∼500 mg/L, with a lower quantification limit of 50 mg/L. The samples and quality control samples were tested three times.

### RNA Extraction and Quantitative Real Time PCR

Cells were collected at 72 h from the HFIM after exposure to the three fosfomycin regimens. RNA expression was then assessed with quantitative real-time PCR as described previously (Sun W. S. et al., 2020). Briefly, Before the quantification of messenger RNA (mRNA) expression by using quantitative real-time PCR, RNA was isolated using the total RNA purificati on kit (GeneMark, Taichung, Taiwan) from bacterial pellets after centrifugation of samples, according to manufacturer’s instructions. After determining the quality and concentration of RNA samples by measuring the OD260/280, 1 μg of RNA was treated with DNase I to remove any contaminated chromosomal DNA and then reverse-transcribed into cDNA using the Power SYBR^®^ Green RNA-to-CT™ 1-Step Kit (Thermo Scientific, Waltham, MA, United States). Quantitative real-time PCR was then performed by applying synthesized cDNA samples, corresponding primer pairs (Supplementary Table 1). In this study, 16S rRNA gene was used as a reference gene to normalize the expression values for the genes being studied.

### *Galleria mellonella* Infection Model

*Galleria mellonella* (Kaide Ruixin Co., Tianjin, China) was utilized to investigate the pathogenicity of resistant mutants which evolved in the HFIM as detailed previously (Lenhard et al., 2015). At the end of the HFIM experiments using three fosfomycin regimens, the resistant strains were collected and stored at −80^°^C prior to virulence assessment. 20 randomly chosen caterpillars 250–350 mg in weight and without gray marks were used in each group. A 10-μL Hamilton syringe was used to inject 10-μL aliquots of the inoculum into the hemocoel of each caterpillar via the last left proleg. Bacterial colony counts were used to confirm consistency of inoculum, and appropriate control arms with caterpillars receiving no injection or an injection of phosphate buffered saline were included. Caterpillars were considered dead when they displayed no movement in response to touch and the number of Caterpillars deaths was recorded every 12 h over 72 h. Virulence assessments were completed in triplicate.

### Statistical Analysis

All statistical analyses were performed with GraphPad Prism, version 8.0 (GraphPad Software, Inc., San Diego, CA, United States). Comparison of the accuracy between the observed and targeted concentration values of fosfomycin using linear regression. One-way ANOVA was used to evaluate the significant differences in the mRNA expression. Survival analyses were performed using Kaplan-Meier survival curves, and significant differences between groups were tested using the log-rank test. *P*-values < 0.05 were considered statistically significant.

## Results

### *In vitro* Susceptibility Testing and Mutational Frequency to Resistance

The results of *in vitro* susceptibility testing and mutational frequency to resistance were listed in Table 1. The MICs of fosfomycin against three clinical isolates were 128 mg/L, while that of linezolid were 2 mg/L. In addition, all organisms were resistant to vancomycin. The mutational frequencies to resistance were 1.91–3.41 × 10^–6^, with fosfomycin at a concentration of 3 × baseline MIC incorporated into the selecting agar. For linezolid, the mutational frequencies to resistance were 1.90–5.38 × 10^–9^.

**TABLE 1:** MICs and MFs of antimicrobial agents against four strains.

Isolates	MIC (mg/L)			MF	
	FOS	LIN	VAN	FOS	LIN
ATCC 51299	64	2	128	2.75 × 10^–6^	5.38 × 10^–9^
NO.1	128	2	>512	3.36 × 10^–6^	1.90 × 10^–9^
NO.2	128	2	>512	1.91 × 10^–6^	3.86 × 10^–9^
NO.3	128	2	>512	3.41 × 10^–6^	2.46 × 10^–9^

*Note: VAN: ≤4 mg/L, susceptible (S); 8–16 mg/L, intermediate (I); ≥32 mg/L, resistant (R). LIN: ≤2 mg/L, susceptible (S); 4 mg/L, intermediate (I); ≥8 mg/L, resistant (R). FOS: ≤64 mg/L, susceptible (S); 128 mg/L, intermediate (I); ≥256 mg/L, resistant (R).*

*Abbreviations: MIC, minimum inhibitory concentration; MF, mutation frequency; FOS, fosfomycin; LIN, linezolid; and VAN, vancomycin.*

### *In vitro* Static-Concentration Time-Kill Study

**FIGURE 1:**
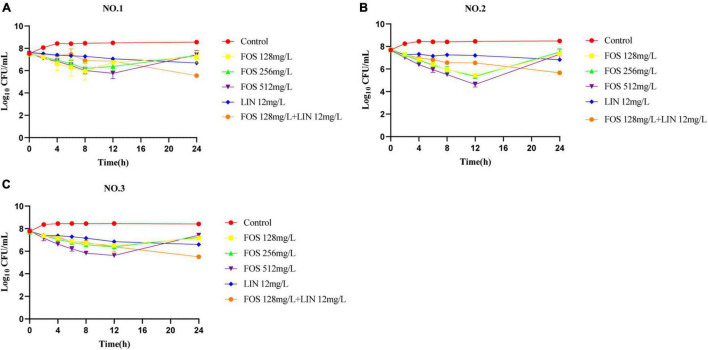
Static-concentration time-kill studies displaying the activity of fosfomycin, linezolid and their combination against vancomycin-resistant *Enterococcus faecium.* NO.1 **(A)**, NO.2 **(B)**, and NO.3 **(C)**. FOS, fosfomycin; LIN, linezolid; Control: no drug.

The pharmacodynamic activities of fosfomycin, linezolid and their combination in SCTK against three strains were depicted in Figure 1. For all strains, fosfomycin alone produced extensive initial killing (1 to 3 log_10_ CFU/mL) within 8–12 h followed by regrowth, although the final population density remained a little lower (∼1 log_10_ CFU/mL) compared to that of the untreated control. Remarkably, for VREfm NO.2, fosfomycin at concentrations of 512 mg/L resulted in maximal bacterial reductions at 12 h of 3 log_10_ CFU/mL (Figure 1B). Linezolid alone showed a steady decline in activity against all three strains over 24 h. Differently, the combination of linezolid and fosfomycin showed bacteriostatic effect on the three strains with ΔlogCFU_0–24_ values of −2.03, −2.01, and −2.30, respectively. The final results indicated that their combined group had additive effects on all three strains, with values of −1.14, −1.17, and −1.09 log_10_ CFU/mL.

### Pharmacokinetic-Pharmacodynamic Analysis

As shown in Supplementary Figure 1, the linear relationship between the observed and targeted concentrations in the first 8 h for all fosfomycin dosing regimens was observed and the correlation coefficient (*R*^2^) was 0.99. To more fully illustrate this good consistency, Supplementary Figure 2 showed the targeted fosfomycin concentration-time profiles overlaid with the mean observed fosfomycin concentrations over 72 h for all dosing regimens.

**FIGURE 2:**
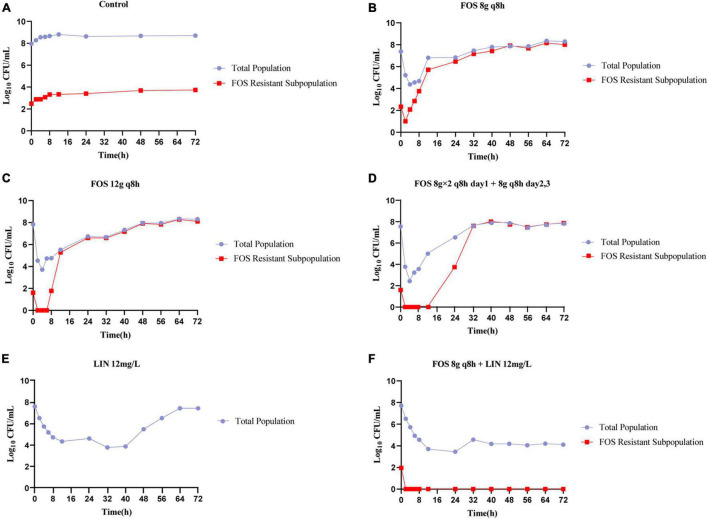
Bacterial counts of the total population and drug-resistant subpopulations following fosfomycin and linezolid in monotherapies and combination studied in the HFIM. Control **(A)**, FOS 8g q8h **(B)**, FOS 12g q8h **(C)**, FOS 8g × 2 q8h day1 + 8g q8h days2,3 **(D)**, LIN 12mg/L **(E)**, and FOS 8g q8h + LIN 12mg/L **(F)**. FOS, fosfomycin; LIN, linezolid; q8h, every 8 h; Control: no drug.

The pharmacodynamic activities of fosfomycin and linezolid as monotherapies or in combination against VREfm NO.2 in the HFIM were shown in Figure 2. The no-treatment control regimen grew well, achieving a bacterial density approaching 1.0 × 10^8^.^55^ CFU/mL by 4 h and resistant subpopulations on agar containing 3× the baseline MIC of fosfomycin also emerged quickly, reaching 1.0 × 10^3^.^74^ CFU/ml by 72 h (Figure 2A). All fosfomycin monotherapy regimens produced rapid initial killing of VREfm NO.2, followed by rapid regrowth close to growth control values, with the emergence of a large number of less-susceptible bacteria. The fosfomycin dose of 8 g q8h achieved bactericidal activity and a maximal bacterial reduction of 3.01 log_10_ CFU/ml after 4 h (Figure 2B), while the 12 g q8h regimen provided bactericidal activity within 2 h and a maximal bacterial reduction of 4.13 log_10_ CFU/ml at 4 h (Figure 2C). There was a significant improvement in bacterial killing for the front-loaded (8 g × 2 q8h) regimen over a short duration, with a maximal reductions of 5.13 log_10_ CFU/mL at 4 h (Figure 2D). The fosfomycin dose of 8 g q8h allowed an increase in the size of the resistant subpopulations above that at the baseline to be seen at 6 h and the resistant subpopulations almost completely replaced the total population by 48 h, while with the 12 g q8h regimen, the resistant subpopulations did not show up until 8 h. Furthermore, the front-loaded (8 g × 2 q8h) regimen resulted in greater killing and the resistant subpopulations went above that at the baseline was delayed until 24 h. Linezolid monotherapy caused a maximal bacterial reductions at 32 h of 3.82 log_10_ CFU/mL, followed by regrowth to near the baseline inoculum by 72 h (Figure 2E). Interestingly, no linezolid-resistant subpopulations were detected in all HFIM experiments. MICs of fosfomycin for all isolates obtained from the fosfomycin monotherapy regimens were >2,048 mg/L, >2,048 mg/L, and 2,048 mg/L, respectively, representing at least an 16-fold MIC elevation.

In contrast to the monotherapies investigated in the HFIM, the combination of linezolid with the lowest dose of fosfomycin was able to achieve extensive killing that was largely maintained for the duration of the experiment (Figure 2F). The combination not only achieved bactericidal activity within 72 h, but also showed significant synergistic effects between 56 h and 72 h. Strikingly, combination therapy completely suppressed all amplification of resistant subpopulations, even though the resistant subpopulations existed at baseline for fosfomycin.

### Relative Quantification of Virulence Gene Expression

**FIGURE 3:**
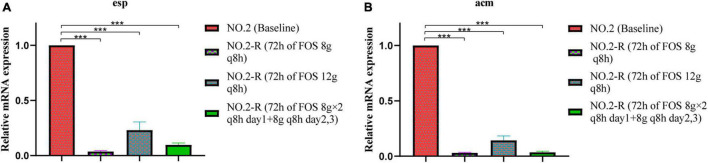
The relative mRNA expression levels of virulence genes **(A)** *esp*, **(B)** *acm* were compared between baseline isolate and fosfomycin-resistant isolates. NO.2-R, isolate resistant to NO.2; FOS, fosfomycin; q8h, every 8 h; “***”, *p*- value ≤ 0.001.

Differences between fosfomycin-resistant strains and the baseline strain were compared for the relative expression of virulence genes (Figure 3). Compared with the baseline isolate, the expression of *esp* in different fosfomycin regimens was significantly down-regulated to 0.036, 0.222, and 0.097 fold (Figure 3A), while that of *acm* was down-regulated to 0.031, 0.141, and 0.034 fold (Figure 3B), respectively. In addition, increasing the exposure of fosfomycin did not result in an additional benefit of reducing the expression of virulence genes, indicating that the exposure of fosfomycin was not positively correlated with the expression of virulence genes.

### *Galleria mellonella* Infection Model

**FIGURE 4:**
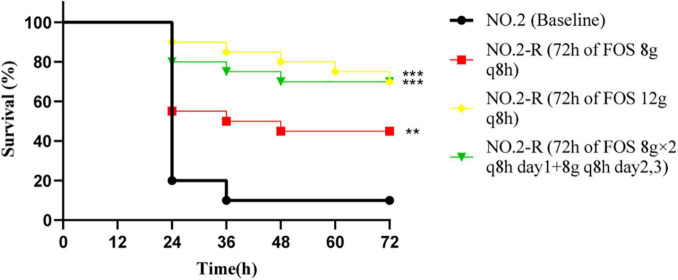
The *Galleria mellonella* infection model was used to explore the difference in pathogenicity between baseline isolate and resistant isolates after three fosfomycin regimens. The significant difference of survival rate at 72 h was analyzed by three resistant isolates and baseline isolate, respectively; NO.2-R, isolate resistant to NO.2; FOS, fosfomycin; q8h, every 8 h;“**”, *p*- value ≤ 0.01; “***”, *p*- value ≤ 0.001.

To determine what impact fosfomycin resistance has on the virulence of the VREfm, we used resistant strains obtained at the 72 h terminal time point of each fosfomycin experiment for inoculation with *G. mellonella* (Figure 4). According to the results of bacterial colony counts, the caterpillars were infected by 4 × 10^6^ CFU/larvae. When comparing the survival rates of different resistant strains with baseline isolate, doses of 12 g q8h and 8 g × 2 q8h resulted in significantly better *G. mellonella* survival relative to the baseline isolate (*G. mellonella* survival ≥ 70% vs. 20% at 72 h), while the 8 g q8h regimen resulted in slightly better survival relative to the baseline isolate (*G. mellonella* survival = 45% vs. 20% at 72 h).

## Discussion

Both SCTK and HFIM results clearly indicated that linezolid combined with fosfomycin exhibited good bactericidal activity against VREfm *in vitro*. In the HFIM, although linezolid monotherapy regimen could achieve a bactericidal effect, it subsequently regenerated to near baseline inoculum. Similarly, Tsuji et al. (2012) found that the front-loaded doses of linezolid provided the maximal bacterial reduction of approximately 3 log_10_ CFU/ml against VREfm in the HFIM *in vitro*. However, it has been reported that the simulated human 600 mg q12h linezolid dosing regimen in a rabbit endocarditis model can only achieve simple bacteriostatic effect (a <3 log reduction in colony counts) against VREfm (Jacqueline et al., 2009). Differences in the antibacterial activity of linezolid *in vitro* and *in vivo* may be related to the “synergistic effect” of the agent with innate host defense cells and/or molecules *in vivo* (Abdelhady and Mishra, 2019). All fosfomycin monotherapy regimens reached their maximum bactericidal effect (3.01 to 5.13 log_10_ CFU/mL) at 4 h, and the maximum bactericidal effect increased with increasing the initial concentration of fosfomycin. This finding indicated that fosfomycin exhibited rapid and concentration-dependent bactericidal activity against VREfm in the early stage. Considering this pharmacodynamic property of fosfomycin, we even used the front-loaded dose that exceeded the scope of clinical trials of fosfomycin to achieve better efficacy. The front-loaded dosing regimen was a strategy to maintain drug efficacy without precipitating drug resistance by giving a high dose of antibiotics in an early short-term treatment followed by a maintenance dose (Zhao et al., 2017). Unfortunately, the front-loaded dose of fosfomycin did not completely eradicate the bacteria. This result may be explained in an *in vitro* bladder infection model, where Abbott et al. found that peak concentrations of up to 1,984 mg/L for a single 3 g dose of fosfomycin or up to 1,129 mg/L for two-doses of fosfomycin were required to inhibit the regeneration of most enterococcus (Abbott et al., 2020). Compared with the monotherapy studied in the HFIM, the combination of linezolid and fosfomycin produced a lasting bactericidal effect on VREfm even at lower dose of fosfomycin. However, the detailed mechanism of action of linezolid combined with fosfomycin remains unclear. Fosfomycin damaged and thinned the cell wall by destroying the outer structure of the bacteria, making it more permeable (Falagas et al., 2016). Linezolid could be considered a synthetic antibiotic of the oxazolidinone class, which inhibits the synthesis of bacterial proteins by binding to rRNA on the 30 s and 50 s ribosome subunits (Hashemian et al., 2018). Therefore, we speculated that fosfomycin may increase the permeability of cell wall and make linezolid enter bacterial cells more easily, thereby playing a continuous bactericidal effect.

In addition to efficacy, we focused more on the ability of combination to inhibit the growth of resistant subpopulations. Understanding the changes in resistant subpopulations is important to guide the rational application of antibiotics and prevent bacterial resistance. The known mechanisms of linezolid resistance in enterococcus were primarily 23S rRNA mutations, and mutations in ribosomal proteins L3, L4, and L22, followed by the transferable resistance genes, including cfr, optrA, and poxtA (Zou and Xia, 2020). Interestingly, no linezolid-resistant subpopulations were found in this study and we suggested that this may be related to the low frequency of enterococcus resistance to linezolid *in vitro* (Drago et al., 2008). Fosfomycin resistance often occured in the treatment of severe infections with high MICs or high bacterial density (Bilal et al., 2018; Rodríguez-Gascón and Canut-Blasco, 2019). Correspondingly, mechanisms associated with fosfomycin resistance in enterococcus have been proposed, including the presence of the *fosB* gene, high-level expression of *fosX* and mutations in the target enzyme *MurA* (Guo et al., 2017; Sun et al., 2017; Zhang et al., 2020). In the HFIM, all fosfomycin regimens failed to inhibit the growth of the resistant subpopulations, and eventually the resistant subpopulations almost completely replaced the total population within 24 h. It was possible that fosfomycin resistance emerged so rapidly because of the high frequency of fosfomycin resistance mutants *in vitro*, as VREfm had a high frequency of fosfomycin resistance mutations of about 10^–6^ in this study. However, Oliva et al. (2014) did not found fosfomycin-resistant strains in guinea pigs that failed fosfomycin treatment in a guinea pig model of foreign body infection. This may be explained by the fact that fosfomycin resistance develops readily *in vitro* but less so *in vivo* (Karageorgopoulos et al., 2012; Falagas et al., 2019). This discordances at least partly be attributed to the function of the immune system *in vivo* (Mei et al., 2015) and the lower likelihood that fosfomycin was selected in acidic environments (Díez-Aguilar and Cantón, 2019). Several HFIM experiments have demonstrated that fosfomycin monotherapy could be ineffective, but in combination with different antimicrobials have shown efficacy against multidrug-resistant Enterococcus, *Escherichia coli*, *Klebsiella pneumoniae*, and *Pseudomonas aeruginosa* clinical isolates (Sime et al., 2016; Drusano et al., 2018; Hemapanpairoa et al., 2019; Portillo-Calderón et al., 2021). Our results have also confirmed this conclusion that the addition of linezolid to fosfomycin eradicated the resistant subpopulations observed with fosfomycin alone against VREfm. Similar to our results, it has been previously observed that another drug in the antibiotic combination may eradicate the subpopulations that are less susceptible to fosfomycin (Broussou et al., 2018; Diep et al., 2018). Furthermore, previous study in our group has found that the combination of linezolid and fosfomycin effectively inhibited the selection of enterococcus resistant mutants by closing each other’s resistance mutation selection windows (Jiang et al., 2021).

Isolates obtained from the fosfomycin monotherapy regimens exhibited high degree of resistance to fosfomycin, with their fosfomycin MICs ≥ 2,048 mg/L. There may be an interaction between fosfomycin resistance and bacterial virulence and more importantly understanding this interaction will help to elucidate the evolution of fosfomycin resistant strains and facilitate the prevention and treatment of serious infections caused by fosfomycin resistant strains. In the present study, the expression of virulence genes (*esp,acm*) and the mortality of *G. Mellonella* decreased with the increase of fosfomycin resistance, suggesting that the virulence of strains decreased with the increase of fosfomycin resistance. This result was also confirmed in a murine model of urinary tract infection, where the virulence of fosfomycin-resistant strains was lower than that of wild-type strains (Pourbaix et al., 2017). In conclusion, the ability of VREfm to resist fosfomycin monotherapy appeared to come at the expense of virulence. We speculated that this compensatory cost may be similar to that previously reported in terms of the possible transfer of virulence-traits and antimicrobial resistance (Lata et al., 2016). However, VREfm is organism of low virulence and low pathogenicity (Puchter et al., 2018; Wada et al., 2020), and the beneficial effect on the clinical efficacy of fosfomycin brought about by the attenuated virulence of their strains is much less than the adverse effect on the clinical efficacy of fosfomycin brought about by the high expression of fosfomycin resistance. Therefore, in the face of severe infections where fosfomycin monotherapy may cause a high degree of resistance, the combination of fosfomycin is recommended.

We acknowledge several limitations in this study. First, only a single isolate of VREfm was studied in the HFIM and might not be representative of all VREfm. To be more universal and representative, more isolates with different MIC values should be studied. Second, the HFIM failed to take account of the host immune response. This made the findings conservative, not only in terms of potential bacterial killing, but also especially in overestimating the likely impact of the emergence of resistance. Meanwhile, in contrast to mammalian models, *G. mellonella* larvae did not possess an adaptive immune system (Cools et al., 2019). Therefore, additional animal studies may be needed in the future to assess the role of the immune system. Furthermore, we only quantitatively examined the related mRNA expression of two virulence genes, but ignored the remaining virulence genes and other potential influencing factors. Taking into account the complex relationship between fosfomycin resistance and virulence, it is necessary to use genomics (Sun Z. et al., 2020; Batool et al., 2021) and other methods for further study. Finally, we would like to emphasize that although the combination regimen of low-dose fosfomycin provided excellent efficacy in VREfm, it is also usually accompanied by potential adverse effects, such as sodium overload and hypokalemia, especially in patients with clinical heart failure (Iarikov et al., 2015; Rodríguez-Gascón and Canut-Blasco, 2019), so clinicians should always monitor the physical health of their patients.

For severe infections with high bacterial load caused by VREfm, even if we used the front-loaded dose that exceeded the scope of clinical trials of fosfomycin, we could not inhibit the amplification of resistant subpopulations. Although the development of fosfomycin resistance was compensated by reducing the virulence of VREfm, the combination of linezolid with fosfomycin was recommended from a clinical efficacy perspective and the combination not only produced more sustained bactericidal effect on the VREfm, but also completely inhibited the amplification of resistant subpopulations. Further clinical studies are warranted to evaluate this promising combination regimen.

## Data Availability Statement

The original contributions presented in the study are included in the article/Supplementary Material; further inquiries can be directed to the corresponding author/s.

## Author Contributions

XH conceived the idea and designed the study. SW performed the study and wrote the manuscript. SW, HL, and JM collected the data and analyzed the results. YP, YY, and YoL provided technical support. NZ, LJ, YnL, and JL revised the manuscript. All authors contributed to read and approved the submitted and final version.

## Conflict of Interest

The authors declare that the research was conducted in the absence of any commercial or financial relationships that could be construed as a potential conflict of interest.

## Publisher’s Note

All claims expressed in this article are solely those of the authors and do not necessarily represent those of their affiliated organizations, or those of the publisher, the editors and the reviewers. Any product that may be evaluated in this article, or claim that may be made by its manufacturer, is not guaranteed or endorsed by the publisher.

## Supplementary Material

Supplementary Figure 1 Relationship between observed and targeted fosfomycin concentrations simulated in the HFIM.

Supplementary Figure 2 The targeted fosfomycin concentration-time profiles (solidlines) overlaid with the average observed fosfomycin concentrations (red circles) for three fosfomycin dosing regimens. FOS, fosfomycin.
