# Supplementary figures and images for: Pharmacodynamics of Linezolid Plus Fosfomycin Against Vancomycin–Resistant Enterococcus faecium in a Hollow Fiber Infection Model
Source: Front Microbiol. 2021 Dec 14;12:779885. doi: 10.3389/fmicb.2021.779885 (PMC8714187; doi:10.3389/fmicb.2021.779885)

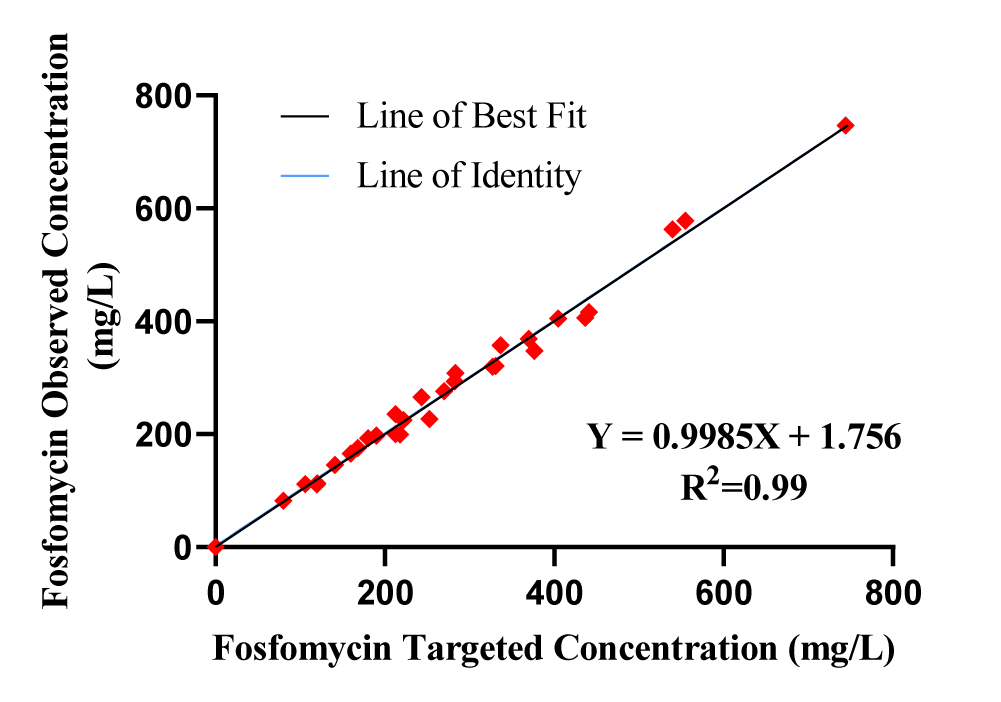

Supplement: Supplementary Figure 1 — Relationship between observed and targeted fosfomycin concentrations simulated in the HFIM. [file Image_1.TIF]

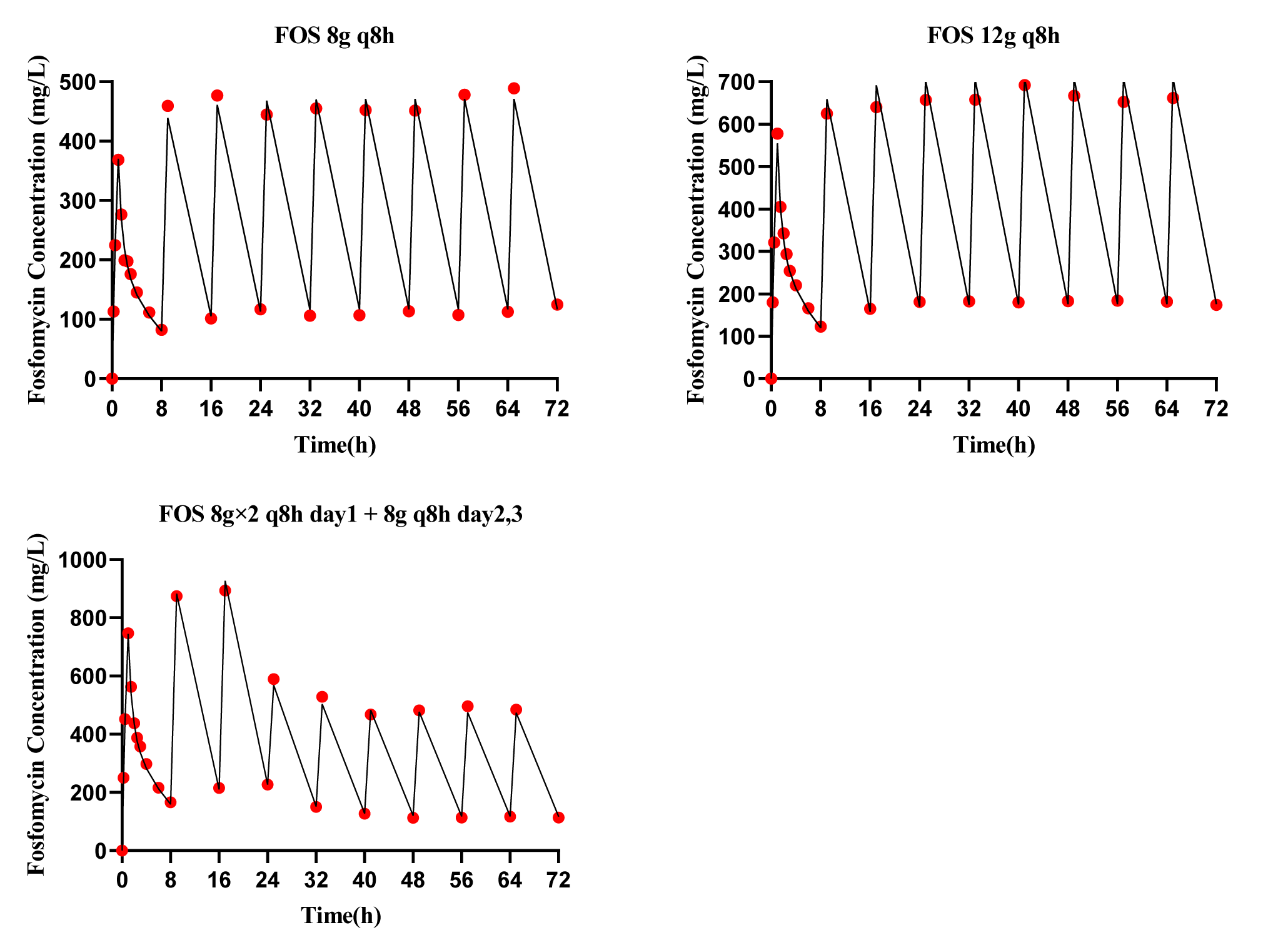

Supplement: Supplementary Figure 2 — The targeted fosfomycin concentration-time profiles (solidlines) overlaid with the average observed fosfomycin concentrations (red circles) for three fosfomycin dosing regimens. FOS, fosfomycin. [file Image_2.TIF]
